# A comparison of off-pump and on-pump coronary bypass surgery in patients with low EuroSCORE

**DOI:** 10.1186/1749-8090-9-105

**Published:** 2014-06-19

**Authors:** Abdulkadir Ercan, Ilker Hasan Karal, Orcun Gurbuz, Gencehan Kumtepe, Tolga Onder, Davit Saba

**Affiliations:** 1 Department of Cardiovascular Surgery, Balikesir University, School of Medicine, Balikesir 10010, Turkey; 2 Department of Cardiovascular Surgery, Samsun Hospital for Education and Research, Ilkadim 55090, Samsun, Turkey; 3 Department of Cardiovascular Surgery, Uludag University, School of Medicine, Bursa, Turkey

**Keywords:** Coronary artery surgery, On-pump, Low-risk, Off-pump

## Abstract

**Background:**

The aim of the present study was to evaluate and compare postoperative short-term, mid-term and long-term outcomes of coronary artery bypass surgery performed with or without cardiopulmonary bypass in patients with a low European System for Cardiac Operative Risk Evaluation score.

**Methods:**

A retrospective analysis of 478 consecutive low risk patients undergoing coronary bypass surgery between January 2002 and December 2007 was performed. Of these patients, 83 cases had undergone on-pump and 395 cases had undergone off-pump coronary bypass surgery. The patients were assessed in terms peri-operative complications, survival, mortality due to cardiac events, need for rehospitalization and repeated coronary revascularization.

**Results:**

There was no significant difference between the two groups in terms of preoperative characteristics, except for chronic obstructive pulmonary disease. The number of distal anastomosis per patient was significantly lower in the off-pump group than in the on-pump group (2.66 ± 0.74 vs. 3.21 ± 0.85, p < 0.001). Early mortality rates were similar in both groups (1.01% for the off-pump group and 1.2% for the on-pump group, p = 0.687). Neurological complications were significantly lower in the off-pump group than in the on-pump group (1.1% vs. 6%, p = 0.01). The mean follow-up period was 80 ± 19.1 months (range, 3–112 months). The need for revascularization during long-term follow-up was 10.1% in the off-pump group and 7.2% in the on-pump group (p = 0.416). The 5-year survival was 95.2 ± 1.1% and 95.5 ± 2.7% in the off-pump and on-pump groups, respectively (p = 0.8), whereas the 7-year survival was 91.9 ± 1.6% and 84.7 ± 6.8% in the off-pump and on-pump groups, respectively (p = 0.274). The 5-year revascularization-free period was 89.5 ± 1.6% and 89.7 ± 3.5% in the off-pump and on-pump groups, respectively (p = 0.785). The 7-year revascularization-free period was 71.1 ± 3.1% and 73.5 ± 7.3% in the off-pump and on-pump groups, respectively (p = 0.075). The 7-year event-free survival was 80.1 ± 2.2% and 73.4 ± 7.3% in the off-pump and on-pump groups, respectively (p = 0.377).

**Conclusions:**

The present study demonstrated that off-pump cardiac surgery had advantages over on-pump cardiac surgery in the short term; however, both interventions had similar mid-term and long-term outcomes, when performed in low-risk patient.

## Background

On-pump coronary bypass surgery is the most frequently used method in coronary artery surgery, accounting for 80% of surgical revascularization in the United States of America [1]. On-pump coronary bypass surgery is associated with higher cardiac, pulmonary, renal, neurological, and bleeding complications, and therefore off*-*pump coronary artery bypass surgery (OPCAB) has gained increased interest since the mid 1990s, as a strategy to protect especially high-risk patients from complications [2-4]. The medical literature contains relatively few reports comparing on-pump to OPCAB in low risk patients. Nathoe et al. [5] reported no significant difference between the two methods regarding cardiac outcomes during a one-year follow-up.

As European System for Cardiac Operative Risk Evaluation (EuroSCORE) as a well-known cardiac surgical risk scoring systems to predict postoperative mortality in patient undergoing cardiac surgery, this retrospective study aimed to compare the postoperative short-, mid- and long-term outcomes of on-pump and OPCAB coronary surgery in low-risk patients according to EuroSCORE evaluation [6].

## Methods

### Patients

All patients with a EuroSCORE of ≤2 who underwent elective isolated coronary surgery at the Uludag University Faculty of Medicine hospital between January 2002 and December 2007 were included. This study was approved by the Uludag Medical Research Ethics committee.

Exclusion criteria were as follows: men aged over 70 and women aged over 65 years old, previous history of cardiac surgery, critical preoperative state (need for inotropic drug support or intra-aortic balloon pumping (IABP), acute renal failure, requiring respiratory support, history of cardiopulmonary resuscitation in the preoperative period), patients with left ventricular ejection fraction under 30% and cases converted to on-pump during the procedure. Preoperative, intraoperative and 30-day postoperative outcomes were retrospectively collected from the hospital records. Postoperative myocardial infarction (MI) was defined as cardiac Troponin I (cTnI) or creatine kinase MB isoenzyme (CKMB) values above five times the normal reference range during the initial 72 hours following coronary artery bypass graft (CABG), when associated with the appearance of new pathological Q-waves or new left bundle branch block (LBBB) [7]. Postoperative acute renal failure was defined as an increase of 100% in serum creatinine. Postoperative pulmonary complication was defined as pleural effusion, atelectasis, phrenic nerve paralysis, diaphragmatic dysfunction, pneumonia, acute respiratory distress syndrome, pneumothorax or chylothorax. Postoperative neurologic complication was defined as any new focal neurologic deficit or change in mental status occurring in the postoperative period.

Long-term follow-up was obtained both from hospital records and phone calls. Current general health status by the New York Heart Association (NYHA) classification, mortality (patient death reported by patients’ relatives or hospital records), late cardiac-related hospitalization (hospital admission for myocardial infarction or heart failure reported by patient or hospital records), repeated angina (hospital visit for angina reported by patient or hospital records), requirements of coronary angiography and repeate revascularization (repeat operation or percutaneous cardiac intervention (PCI) since the operation reported by patient or hospital records) were determined.

### Surgical technique

As a clinical routine method all patients were premedicated with subcutaneous morphine sulfate (0.1 mg/kg). Anesthetic induction was performed with fentanyl (5 μg/kg), midazolam (0.1 mg/kg), and vecuronium (0.1 mg/kg). Maintenance of anesthesia was provided by fentanyl (0.5-1 μg/kg), vecuronium (0.1 mg/kg) and 0.5-1 minimum alveolar anesthetic concentration (MAC) of isoflurane. Classic median sternotomy, left internal thoracic artery harvesting and other conduits preparation were performed by a standard technique. Heparin was administered to keep the activated clotting time (ACT) greater than 300 seconds during OPCAB and greater than 450 seconds during on-pump coronary bypass surgery.

Octopus II, III or IV (Medtronic Inc., Minneapolis, MN, US) were used as local myocardium stabilizer. Proximal coronary clamping of all target vessels was performed by Mueller atraumatic vascular clamps (0.5 Newton), distal occlusion was never performed. Filtered room air insufflations (<5 L/min) was employed to provide better visibility during anastomosis. All proximal anastomoses were performed under single side clamping, using 6/0 prolene sutures. Distal anastomoses were performed by end-to-side or side-to-side techniques with a running 7/0 prolene suture. During distal anastomosis and reperfusion, 20% mannitol (2 mL/kg) was routinely administered. At the end of surgery heparin was neutralized by protamine providing that ACT would be 150 to 180 seconds. The patients were transferred to the intensive care unit and were connected to a volume-controlled ventilator. In the early postoperative period (6–8 hours), low molecular-weight heparin and 100 mg acetylsalicylic acid was commenced routinely.

In patients undergoing on-pump coronary bypass surgery, cardiopulmonary bypass (CPB) was established with an ascending aortic arterial cannula and a right atrial two stage venous cannula, using a membrane oxygenator and a roller pump. All patients were cooled to 28-30°C. After aortic cross-clamping, anterograde cold crystalloid cardioplegic solution (15 mL/kg) was initially administered. Mean arterial blood pressure was maintained in the range of 60–90 mmHg. Diastolic cardioplegic arrest was maintained with cold blood-cardioplegic solution at a ratio of 1:4 at 20 minutes intervals, and warm blood cardioplegia (hot shot) was given for 5 min before the cross clamp release. Distal anastomoses were performed by end-to-side or side-to-side techniques with a running 7/0 prolene suture. Proximal anastomoses were performed using 6/0 prolene suture during the heating period with the assistance of ascending aortic side-clamp. After the completion of CPB and cannula removal, heparin was neutralized with protamine providing an ACT less than 150 seconds. Acetylsalicylic acid at a dose of 100 mg and low molecular-weight heparin was initiated on the postoperative 24th hours.

All the patient were discharged under asetilsalisilic asid therapy. Moreover all of them were under single antiagregant-drug therapy at the time of phone call.

### Statistical analysis

Descriptive statistics were expressed as mean ± standard deviation. The Shapiro-Wilk test was used to test normality of continuous variables. The non-parametric Mann–Whitney *U* test was used for intergroup comparisons. Categorical variables were compared using Pearson’s Chi-square and Fisher’s exact chi-square tests. A z-test was used to compare proportions. Survival analysis, angina-, MI-, stroke-free period, and revascularization-free period were compared using the Kaplan-Meier analysis and the Log-Rank test. A p value <0.05 was considered statistically significant.

## Results

The rate of conversion to CPB was 2.5% including 22 patients which were excluded from the study and eventually data on 478 patients were extracted and follow-ups were completed. Of these patients, 395 (82.6%) were in the OPCAB group and 83 (17.4%) were in the on-pump group. Baseline patient characteristics are shown in Table 1. Chronic obstructive pulmonary disease (COPD) prevalence was found significantly higher in on-pump group than OPCAB group.

**Table 1:** Base-line characteristics of the study groups

**Characteristics**	**OPCAB group (n = 395)**	**On-pump group (n = 83)**	**p**
**Age (years)**	54.7 ± 7.27	54.02 ± 7	0.353
**Male Sex**	358 (90.6)	75 (90.4)	0.939
**BMI (kg/m**^**2**^**)**	27.59 ± 3.24	27.06 ± 3.36	0.194
**EuroSCORE (add)**	1.03 ± 0.82	0.86 ± 0.80	0.090
**EuroSCORE (log)**	1.24 ± 0.30	1.17 ± 0.30	0.053
**CCA Class 1**	5 (1.3)	0 (0.0)	0.024*
**Class 2**	75 (19.0)	11 (13.3)	0.173
**Class 3**	223 (56.5)	52 (62.7)	0.291
**Class 4**	92 (23.3)	20 (24.1)	0.876
**Recent MI (<90 days)**	53 (13.4)	12 (14.5)	0.802
**PCI**	17 (4.3)	6 (7.2)	0.261
**Current smoker**	294 (74.4)	63 (75.9)	0.779
**Family history**	206 (52.2)	52 (62.7)	0.081
**Diabetes mellitus**	106 (26.8)	24 (28.9)	0.699
**Dyslipidemia**	222 (56.2)	49 (59.0)	0.636
**CRF**	1 (0.3)	0 (0.0)	0.646
**Hypertension**	209 (52.9)	47 (56.6)	0.537
**CVD**	1 (0.3)	2 (2.4)	0.079
**COPD**	3 (0.8)	4 (4.8)	0.020*
**PVD**	8 (2)	2 (2.4)	0.687
**Carotid artery disease**	4 (1)	1 (1.2)	1.000
**LMCA**	36 (9.1)	8 (9.6)	0.881
**One Vessel disease**	27 (6.8)	2 (2.4)	>0.05
**Two Vessel disease**	132 (33.4)	17 (20.5)	0.010*
**Three Vessel disease**	236 (59.7)	64 (77.1)	0.010*
**Normal left ventricular function**	303 (76.7)	70 (84.3)	0.127
**Moderate left ventricular function**	92 (23.3)	13 (15.7)	0.010*

*Statistically significant difference.

Values are presented as mean ± standard deviation or number (%), where appropriate. **OPCAB**, off-pump coronary artery bypass surgery; **CPB**, cardiopulmonary bypass; **BMI**, body mass index; **EuroSCORE**, European System for Cardiac Operative Risk Evaluation (add: additive, log: logarithmic); **CCA**, Canadian Class of Angina; **MI**, myocardial infarction; **PCI**, percutaneous coronary intervention; **CRF**, chronic renal failure; **CVD**, cerebrovascular disease; **COPD**, chronic obstructive pulmonary disease; **PVD**, peripheral vascular disease; **LMCA**, left main coronary artery**; LVEF**, left ventricular ejection fraction.

The rate of patients with three-vessel disease was significantly lower in the OPCAB group (59.7% vs. 77.1%, p = 0.01); however, two-vessel disease was significantly higher in OPCAB group (33.4% vs. 20.5%, p = 0.01). Moreover, OPCAB group included significantly more patients with moderate left ventricular function than on-pump group (23.3% vs. 15.7%, p = 0.01).

Evaluation of the intraoperative and postoperative outcomes revealed that duration of surgery and duration of ventilation period were significantly shorter in the OPCAB group. However, no differences were observed between the groups in terms of duration of intensive care unit and hospital stay and early mortality. The needs for intraoperative inotropic agents and IABP were significantly lower in the OPCAB group. Moreover, the need for postoperative inotropic agents was lower in the OPCAB group. The amount of 24 hours drainage and the amount of consumed blood products were significantly lower in the OPCAB group. However, re-operation due to bleeding was found significantly more common in the OPCAB group. Neurological and pulmonary complications were significantly more common in the on-pump group. No difference was noted between the groups in terms of the rates of postoperative MI, mediastinitis and renal complications (Tables 2 and 3).

**Table 2:** Intraoperative characteristics of the study groups

	**OPCAB group**	**On-pump group**	**p**
**Number of grafts**	2.58 ± 0.67	3.16 ± 0.79	<0.001*
**Number of distal anastomoses**	2.66 ± 0.74	3.21 ± 0.85	<0.001*
**Grafted Vessels**			
Proximal LAD	0	1 (1.2)	0.174
Mid LAD	30 (7.6)	4 (4.8)	0.371
Distal LAD	366 (92.7)	80 (96.4)	0.217
D1	136 (34.4)	36 (43.4)	0.123
D2	23 (5.8)	3 (3.6)	0.596
CxOM1	177 (44.8)	57 (68.7)*	<0.001*
CxOM2	91 (23.0)	26 (31.3)	0.110
RCA	67 (17.0)	25 (30.1)	0.280
RPD	148 (37.5)	28 (33.7)	0.521
RPL	17 (4.3)	6 (7.2)	0.261
**Duration of surgery (min)**	208.49 ± 45.2	291.32 ± 59.1	<0.001*
**Intraoperative inotropic**	23 (5.9)	29 (34.9)	<0.001*
**Intraoperative IABP**	0	2 (2.4)	0.030*
**Intraoperative MI**	0	1 (1.2)	0.174
**Intraoperative arrhythmia**	3 (0.8)	2 (2.4)	0.209
**LIMA-LAD**	385 (97.5)	82 (98.8)	0.698
**RIMA**	1 (0.3)	6 (7.2)	<0.001*
**Radial artery**	39 (9.9)	8 (9.6)	0.948
**Endarterectomy**	2 (0.5)	3 (3.6)	0.039*

*Statistically significant difference.

Values are presented as mean ± standard deviation or number (%), where appropriate. OPCAB, off-pump coronary artery bypass surgery; CABG, coronary artery bypass graft.

**Table 3:** Early postoperative outcomes of the study groups

	**OPCAB group**	**On-pump group**	**p**
**Duration of hospital stay (days)**	4.9 ± 2.4	6.3 ± 3.6	0.786
**Duration of ventilation (min)**	350.67 ± 222	435.92 ± 343	0.005*
**Duration of intensive care unit (h)**	28.44 ± 18.5	38.57 ± 69.7	0.069
**Drainage (mL/24 h)**	455.74 ± 216.9	567.83 ± 303.8	0.001*
**Blood (unit)**	0.16 ± 0.5	1.14 ± 1.09	<0.001*
**TDP (unit)**	0.81 ± 1.3	3.8 ± 2.32	<0.001*
**Postoperative inotropic agent**	26 (6.6)	28 (33.7)	0.010*
**Postoperative IABP**	1 (0.3)	2 (2.4)	0.079
**Postoperative MI**	5 (1.3)	3 (3.6)	0.147
**Postoperative AF**	39 (9.9)	7 (8.4)	0.174
**Postoperative neurological complication**	4 (1.1)	5 (6.0)	0.010*
**Pulmonary complication**	12 (3.1)	7 (8.4)	0.031*
**Renal complication**	1 (0.3)	0 (0.0)	1.000
**Mortality (<30 days)**	4 (1.01)	1 (1.2)	0.687
**Mediastinitis**	8 (2)	1 (1.2)	1.000
**Re-operation**	7 (1.9)	4 (4.8)	0.698
**Re-operation due to bleeding**	3 (8.0)	1 (1.2)	0.018*

*Statistically significant difference.

Values are represented as mean ± standard deviation and number (%), where appropriate. OPCAB, off-pump coronary artery bypass surgery; CABG, coronary artery bypass graft.

The number of distal anastomosis was 2.66 ± 0.74 in the OPCAB group and 3.21 ± 0.85 in the on-pump group (p < 0.001). Grafting of the first obtuse margin (OM1) artery was significantly more frequent in the on-pump group than in the OPCAB group (68.7% vs. 44.8%%, p < 0.001). However, there were no significant difference in terms of grafting of the other posterior surface coronary arteries, including second obtuse marginal (OM2) artery, right posterior descending (RPD) artery, and right posterolateral (RPL) artery between groups*.* The left internal mammary artery (LIMA) was anastomosed to the left anterior descending artery (LAD) in 97.5% of the patients in the OPCAB group and in 98.8% of the patients in the on-pump group (p = 0.698). The radial artery was used in 9.9% of the patients in the OPCAB group and in 9.6% of the patients in the on-pump group (p = 0.948). However, the right internal mammary artery (RIMA) was more commonly anastomosed in the on-pump group (0.3% vs. 7.2%, p < 0.001). Coronary endarterectomy was performed significantly more in on-pump group than in OPCAB group (3.6% vs. 0.5%, p < 0.039).

The mean follow-up duration was 82.9 ± 19.1 months (range, 3–112 months) in the OPCAB group, whereas it was 67.8 ± 13.3 months (range, 6–85 months) in the on-pump group (p = 0.0). The 5-year survival rates were similar in both groups (95.2 ± 1.1% for the OPCAB and 95.5 ± 2.7% for the on-pump group, p = 0.8). The 7-year survival rates were also similar in both groups (91.9 ± 1.6% for the OPCAB group and 84.7 ± 6.8% for the on-pump group, p = 0.274).

Percutaneous coronary re-intervention was performed in 36 (9.1%) patients in the OPCAB group and in 6 (7.2%) patients in the on-pump group (p = 0.555). Re-operation for coronary bypass was performed in 4 (1%) patients in the OPCAB group, but was not performed in the on-pump group (p = 0.357). The re-intervention-free periods were similar in both groups. The 5-year re-intervention-free period was 89.5 ± 1.6% and 89.7 ± 3.5% in the OPCAB and On-pump groups, respectively (p = 0.785). The 7-year re-intervention-free period was 71.1 ± 3.1% in the OPCAB group and 73.5 ± 7.3% in the On-Pump group (p = 0.075). Evaluation of event-free (angina, MI and stroke) period during the follow-up period revealed that the 5-year event-free period was 90.1 ± 1.5% and 87.9 ± 3.87% in the OPCAB and on-pump groups, respectively, whereas the 7-year event-free period was 80.1 ± 2.2% in the OPCAB group and 73.4 ± 7.3% in the On-Pump group (p = 0.377).

Evaluation of the long-term general health status of the patients revealed that 96.2% of the patients in the OPCAB group and 94% of the patients in the on-pump group defined their health status better as compared to their health status in the preoperative period; the groups showed no difference in this respect (p = 0.424). During long-term follow-up, 10 (2.5%) patients in the OPCAB group and 2 (2.4%) patients in the on-pump group died due to cardiac reasons.

## Discussion

Numerous meta-analyses and randomised trials comparing OPCAB with conventional CABG revealed significantly decreased blood product transfusion [8-12], inotrope requirement [8,9,13], stroke incidence [14,15], postoperative atrial fibrillation [8,9,13], myocardial injury [9,10,15,16], ventilation [8,13] and operation time [8,10], and also İCU (intensive care unit) [13] or hospital stay [8,13,17] in the OPCAB groups. However, these reports did not focus on low-risk patients in both short and long term, as the present study did.

Nathoe et al. [5], in a multicenter randomized trial performed on low-risk patients reported significantly shorter operation time in the OPCAB group than the CPB group. This study also revealed significantly lower duration of surgery in the OPCAB group than the on-pump group (208 min vs. 291 min, p < 0.001).

The amount of drainage in the first 24 hours and the supply of blood products were higher in the on-pump group, consistent with the literature data, which may be explained by well-known adverse effects of extracorporeal circulation on coagulation systems. Nevertheless, the need for re-operation due to bleeding was significantly higher in the OPCAB group than in the CPB group (8% vs. 1.2%, p = 0.018), inconsistent with the prior meta-analysis revealing lower re-operation for bleeding in the OPCAB group [8,9,15]. Our opposite finding may be explained by that fact that the excessive bleeding occurring after OPCAB may not be associated with CPB-related coagulation problems and should be treated surgically, unless ACT is too high.

Wijeysundera et al. [9] in their meta-analysis, comparing OPCAB with on-pump CABG, reported that OPCAB cause less myocardial damage. Several randomized studies also revealed significantly lower myocardial enzymes levels in the OPCAB groups than the CPB groups [10,12,15,16]. İn the present study, the frequency of postoperative MI was found similar in both groups, but the need for inotropic agent and IABP counterpulsation due to low cardiac output was significantly higher in the on-pump group. After all, the OPCAB group included significantly more patients with moderate left ventricular function than on-pump group. These findings indicate that the OPCAB technique caused less myocardial damage when compared with the CPB technique, even in low risk CABG patients.

No significant difference was found between the OPCAB and CPB groups in terms of postoperative AF frequency (9.9% vs. 8.4%, p = 0.174). These finding supports recent report conducted by Nature et al. in low risk CABG patients [5]. However, since continuous monitoring for AF was not performed in the present study, short-lasting AF attacks might have been overlooked.

Neurological complications are generally considered as the most important coronary surgery related morbidity because of their contribution to mortality, effects on patient’s quality of life and socio-economic outcomes [18,19]. Moreover, mortality rate has been reported 10%-21% in patients developed major neurologic deficit [18,20,21]. Studies have led to the thought that CPB and related factors are the most important sources of neurological complications [22,23]. In their meta-analysis, Edelman et al. [14] reported stroke rates to be 0.38% in the OPCAB group, in which aortic manipulation was not performed, and significantly lower than the conventional group (1.87%). Consistently, the present study detected significantly more neurological complications in the on-pump group than the OPCAB group (1,1% vs. 6%, p = 0.01). These findings revealed that even in low-risk patients CPB causes more neurological complications than OPCAB.

Cheng’s [8] meta-analysis revealed significantly shorter ventilatory support and lesser pulmonary infections in the OPCAB group as compared to the conventional CABG group. İn the present study pulmonary complications were observed in 12 (3.1%) patients in the OPCAB group and in 7 (8.4%) patients in the on-pump group (p = 0.031). However, this finding might be affected by significantly more frequency of COPD in the on pump group than the OPCAB group (Table 1).

A mild impairment may be observed in the renal functions in the majority of patients undergoing conventional coronary surgery and this may progress to renal failure requiring dialysis in 1%-5% of the patients, being more frequent in those previously having partially impaired renal function and heart failure [24-26]. The meta-analysis reported by Wijeysundera [9] and Reston [15] demonstrated that impaired renal functions are less observed in the OPCAB procedure as compared to the conventional method. However, Cheng [8] found no significant difference for renal dysfunction, in a meta-analysis comparing OPCAB with conventional CABG. In the present study focusing on low risk patients only one (0.3%) OPCAB patient developed acute renal failure while none of CPB patients did so (p = 0.646).

Graft patency is considered one of the important measures in evaluating the coronary surgery procedures. In recent randomized studies, Puskas et al. [17] and Angelini et al. [27] reported similar graft patency in the conventional and OPCAB groups; however, Khan et al. [12] reported lower patency in the OPCAB group. A meta-analysis of prospective randomized trials comparing off-pump with on-pump coronary graft patency revealed an increased risk of graft occlusion in the OPCAB group [28]. The present study revealed similar late re-intervention rate in the OPCAB and CPB groups (10.1% vs. 7.2%, p = 0,416), in long-term (Table 4).

**Table 4:** Long-term outcomes of the study groups

	**OPCAB group**	**On-pump group**	**p**
**Mean follow *- *** **up time (mounth)**	82.9 ± 19.1	67.8 ± 13.3	0.0
**Cardiac re-hospitalization**	85 (21.5)	13 (15.7)	0.316
**Late cardiac mortality**	10 (2.5)	2 (2.4)	0.945
**Late all cause mortality**	30 (7.6)	2 (2.4)	0.086
**Health status**			
Poor	15 (3.8)	5 (6.0)	0.424
Good-better	380 (96.2)	78 (94.0)	0.424
**Effort test**			
Negative	141 (35.7)	23 (27.7)	0.275
Positive	30 (7.6)	5 (6.0)	0.269
**Angina**	90 (22.8)	14 (16.8)	0.235
**Coronary angiography**			
Normal	27 (6.8)	5 (6.0)	-
Pathological	76 (19.2)	12 (14.4)	0.547
**Late intervention**			
Absent	355 (89.9)	77 (92.8)	0.369
Stent	36 (9.1)	6 (7.2)	0.555
Redo-CABG	4 (1.0)	0 (0.0)	0.357
Cumulative late reintervention	40 (10.1)	6 (7.2)	0.416

Values are represented as mean ± standard deviation and number (%), where appropriate. OPCAB, off-pump coronary artery bypass surgery; CABG, coronary artery bypass graft; Cumulative late reintervention = CABG + Stent.

A meta-analysis of 37 randomized trials reported by Cheng et al. [8] found significantly lower distal anastomosis number in the OPCAB groups than in the CPB groups. İn the present study, the number of grafts and the number of distal anastomoses per patient were detected significantly lower in the OPCAB group (Table 2). However, these findings may be correlated with the lower number of diseased vessels in OPCAB group, as mentioned before (Table 1). Moreover, long-term follow-up did not revealed difference between two techniques, in term of cardiac re-interventions and cardiac mortality (Table 4).

Takagi et al. [29] reported that OPCAB increases late all-cause mortality as compared to conventional group, due to lower revascularization rate and graft patency in OPCAB patients. The present study showed that the 5- and 7-year survival rates were similar in the OPCAB and in the on-pump groups and no difference was observed between the groups in the long-term regarding the event-free period such as need for re-surgery, angina, MI and stroke. The late all-cause mortality rate between groups was similar (7.6% vs. 2.4%, p = 0.080).

## Conclusion

The results of this study conducted in low risk patients support OPCAB’s favorable effects on blood product need, duration of surgery, neurological complications rate, length of intensive care unit stay, postoperative inotropic agent need, frequency of pulmonary complications and duration of ventilation, during the short-term. Moreover, mid- and long-term outcomes revealed that cardiac-related mortality, need for re-intervention and angina-free period were similar to on-pump technique. Despite the advantages of the OPCAB in the short-term, this method was found not be superior to the on-pump method in the mid- and long-terms, in low risk patients. Better short-term data and similar long-term outcomes appears to support reliability and efficacy of the OPCAB method in low risk patient.

The present study has some limitations includin*g* its retrospective design, single-center site and relatively small sample size. Therefore, its result should be cautiously interpreted. Larger prospective randomized, multicenter trials may help define the optimal management of low risk CABG patients.

## Abbreviations

ACT: Activated clotting time; AF: Atrial fibrillation; CABG: Coronary artery bypass graft; CKMB: Creatine kinase MB isoenzyme; COPD: Chronic obstructive pulmonary disease; CPB: Cardiopulmonary bypass; cTnI: Cardiac Troponin I; EuroSCORE: European System for Cardiac Operative Risk Evaluation; h: hour; IABP: İntra-aortic balloon pumping; LAD: Left anterior descending artery; LBBB: Left bundle branch block; LIMA: Left internal mammary artery; MAC: Minimum alveolar anesthetic concentration; MI: Myocardial infarction; NYHA: New York Heart Association; OPCAB: Off*-*pump coronary artery bypass surgery; PCI: Percutaneous cardiac intervention; RIMA: Right internal mammary artery; RPD: Right posterior descending artery; RPL: Right posterolateral artery; OM1: First obtuse marginal artery; OM2: Second obtuse marginal artery; TİA: Transient ischemic attack.

## Competing interests

The authors declare that they have no competing interests.

## Authors’ contributions

IHK and DS designed the study. IHK, TO and GK carried out studies searching and performed the eligibility assessments. AE and OG evaluated the qualities of the included studies and carried out data extracting. AE, İK and OG analyzed and interpreted the data. OG drafted the manuscript. AE made critical revision of the manuscript for important intellectual content. All authors read and approved the final manuscript.
